# Impaired CO_2_ sensitivity of astrocytes in a mouse model of Rett syndrome

**DOI:** 10.1113/JP270369

**Published:** 2015-06-17

**Authors:** Egor Turovsky, Anastassios Karagiannis, Ana Paula Abdala, Alexander V Gourine

**Affiliations:** 1 Centre for Cardiovascular and Metabolic Neuroscience, Department of Neuroscience, Physiology and Pharmacology, University College LondonGower Street, London, WC1E 6BT, UK; 2 Institute of Cell Biophysics, Russian Academy of SciencesPushchino, Russia; 3 School of Physiology & Pharmacology, University of BristolBristol, BS8 1TD, UK

## Abstract

Rett syndrome, a prototypical neurological disorder caused by loss of function of the transcriptional regulator methyl-CpG-binding protein 2 (MeCP2) gene, is associated with a severely disordered breathing pattern and reduced ventilatory CO_2_ sensitivity. In a mouse model of Rett syndrome (*MeCP2* knockout), re-introduction of the *MeCP2* gene selectively in astrocytes rescues normal respiratory phenotype. In the present study we determined whether the metabolic and/or signalling functions of astrocytes are affected by testing the hypotheses that in conditions of MeCP2 deficiency, medullary astrocytes are unable to produce/release appropriate amounts of lactate or detect changes in /[H^+^], or both. No differences in tonic or hypoxia-induced release of lactate from the ventral surface of the medulla oblongata or cerebral cortex in brain slices of *MeCP2*-knockout and wild-type mice were found. In brainstem slices of wild-type mice, respiratory acidosis triggered robust elevations in [Ca^2+^]_i_ in astrocytes residing near the ventral surface of the medulla oblongata. The magnitude of CO_2_-induced [Ca^2+^]_i_ responses in medullary astrocytes was markedly reduced in conditions of MeCP2 deficiency, whereas [Ca^2+^]_i_ responses to ATP were unaffected. These data suggest that (i) metabolic function of astrocytes in releasing lactate into the extracellular space is not affected by MeCP2 deficiency, and (ii) MeCP2 deficiency impairs the ability of medullary astrocytes to sense changes in /[H^+^]. Taken together with the evidence of severely blunted ventilatory sensitivity to CO_2_ in mice with conditional *MeCP2* deletion in astroglia, these data support the hypothesis of an important role played by astrocytes in central respiratory CO_2_/pH chemosensitivity.

**Key points:**

- Rett syndrome is a prototypical neurological disorder characterised by abnormal breathing pattern and reduced ventilatory CO_2_ sensitivity. Medullary astrocytes are a crucial component of central CO_2_/pH chemosensitivity.
- This study tested the hypotheses that methyl-CpG-binding protein 2 (MeCP2) deficient medullary astrocytes are (i) unable to produce/release appropriate amounts of lactate, and/or (ii) unable to sense changes in 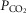/[H^+^].
- We found no differences in tonic or hypoxia-induced release of lactate from the ventral surface of the medulla oblongata or cerebral cortex between *MeCP2*-knockout and wild-type mice.
- Respiratory acidosis triggered robust [Ca^2+^]_i_ responses in wild-type astrocytes residing near the ventral surface of the medulla oblongata. CO_2_-induced [Ca^2+^]_i_ responses in astrocytes were dramatically reduced in conditions of MeCP2 deficiency.
- These data suggest that (i) ‘metabolic’ function of astrocytes in releasing lactate into the extracellular space is not affected by MeCP2 deficiency, and (ii) MeCP2 deficiency impairs the ability of medullary astrocytes to sense changes in 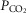/[H^+^].

## Introduction

Astrocytes provide neurones with structural and metabolic support. They are well positioned to respond rapidly to changes in chemical composition of the blood supplying the brain as well as to sense metabolic demands of the neuronal networks acting as an essential cellular component of the neurovascular interface. The astrocyte–neurone lactate shuttle hypothesis proposes that astrocytes support neuronal activity by supplying neuronal networks with metabolic substrate in the form of lactate (Magistretti *et al*. 1993, 1994; Pellerin & Magistretti, 1994). In low 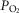 conditions (hypoxia), lactate production by astrocytes increases and appears to be essential for the recovery of synaptic function during re-oxygenation (Schurr *et al*. 1997*a*,*b*,*c*). Recent evidence suggests that astrocytes play an important role in the central nervous mechanisms controlling breathing. Astrocytes residing at and near the ventral surface of the medulla oblongata are intrinsically sensitive to changes in parenchymal 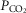/[H^+^] and during hypercapnia contribute to the adaptive increases in breathing (Gourine *et al*. 2010).

Mutations in the human transcriptional regulator methyl-CpG-binding protein 2 (MeCP2) gene leading to loss or reduction of protein function cause a neurodevelopmental disorder called Rett syndrome (Amir *et al*. 1999). Severely arrhythmic breathing which includes frequent apnoeas, hypopneas and periods of hyperventilation is a hallmark of this prototypical neurological disorder (Southall *et al*. 1988; Viemari *et al*. 2005; Ramirez *et al*. 2013). The respiratory dysrhythmia often results in hypoxaemia and hypocapnia (De Felice *et al*. 2014). In animal (mouse) models, MeCP2 deficiency is associated with significantly depressed ventilatory sensitivity to CO_2_, especially in response to mild and moderate levels of hypercapnia (Zhang *et al*. 2011; Bissonnette *et al*. 2014). MeCP2 deficiency also significantly increases the CO_2_ apnoeic threshold (Toward *et al*. 2013). Conversely, ventilatory responses to hypoxia appear to be enhanced (Bissonnette & Knopp, 2006; Voituron *et al*. 2009; Bissonnette *et al*. 2014), while a higher level of inspired oxygen worsens the occurrence of periodic breathing in mice with reduced MeCP2 function (Bissonnette & Knopp, 2008). Interestingly, in the affected individuals and animal models, regularity of breathing is improved by supplemental CO_2_ provided in the inspiratory gas mixture (Smeets *et al*. 2006; Bissonnette & Knopp, 2008).

*MeCP2* is expressed in astrocytes (Yasui *et al*. 2013; Forbes-Lorman *et al*. 2014) and loss of *MeCP2* function causes critical astrocytic defects (Okabe *et al*. 2012). There are *in vitro* data showing that the function of wild-type neurones is compromised when they are co-cultured with MeCP2-deficient astrocytes (Ballas *et al*. 2009; Maezawa *et al*. 2009; Maezawa & Jin, 2010). Earlier reports highlighted the importance of astroglial dysfunction in the development of Rett syndrome and demonstrated that re-expression of *MeCP2* selectively in astrocytes rescues normal respiratory pattern in MeCP2 deficient mice (Lioy *et al*. 2011). Since astrocytes located at and near the ventral surface of the medulla oblongata contribute in a significant manner to the mechanisms of central respiratory CO_2_ chemosensitivity, we proposed that impaired astroglial CO_2_ sensitivity could underlie the respiratory phenotype observed in Rett syndrome.

In this study we first determined whether the ‘metabolic’ function of astrocytes is compromised by MeCP2 deficiency by measuring the amount of lactate released by the brain slices of *MeCP2*-null and wild-type male mice. Then we determined whether Ca^2+^ signalling and CO_2_/pH sensitivity of medullary astrocytes are affected in the absence of MeCP2 function.

## Methods

All the experiments were performed in accordance with the European Commission Directive 2010/63/EU (European Convention for the Protection of Vertebrate Animals used for Experimental and Other Scientific Purposes) and the UK Animals (Scientific Procedures) Act (1986).

### *MeCP2*-null mice

In total 28 *MeCP2*-null and 26 littermate wild-type male mice (65–80 days old) were used for the experiments. *MeCP2* is an X-linked gene subject to random X inactivation (Braunschweig *et al*. 2004) and *MeCP2* mutant heterozygous females show mosaicism. Since phenotype depends on both cell-autonomous and non-cell-autonomous mechanisms (Braunschweig *et al*. 2004), only male mice were used in the current study. We used two *MeCP2*-null strains: B6.129P2(C)-*MeCP2^tm1.1Bird^*/J (*Bird, n* = 26) as a model of a complete *MeCP2* knockout (Guy *et al*. 2001), and *MeCP2^tm1.1Jtc^*/SchvJ (*Coyle, n* = 2), which carries one of the most common mutations that affect humans where a non-functional truncated MeCP2 protein transcript is present (Lawson-Yuen *et al*. 2007). The former was maintained on a C57BL/6J background and the latter on a mixed background ∼87–93% C57BL/6J and ∼13–6% 129S6/SvEvTac. Null males were obtained from breeding heterozygous females to wild-type males. Heterozygous *MeCP2^tm1.1Bird^* females were obtained from The Jackson Laboratory (Bar Harbor, Maine, USA). Their offspring were genotyped using standard PCR, and primer sequences were as follows: common (AAA TTG GGT TAC ACC GCT GA), mutant reverse (CCA CCT AGC CTG CCT GTA CT) and wild-type reverse (CTG TAT CCT TGG GTC AAG CTG). Heterozygous *MeCP2^tm1.1Jtc^* were donated by Dr Laura R. Schaevitz (Tufts University). Their offspring were genotyped using Sanger sequencing, and primer sequences were as follows: *MeCP2* forward (AGG TGG GAG ACA CCT CCT TG) and *MeCP2* reverse (GAC CTG GGC AGA TGT GGT AG).

### Slice preparation

Mice were terminally anaesthetised with a halothane inhalation overdose. The brains were removed and placed in ice cold artificial cerebrospinal fluid (aCSF) containing 124 mm NaCl, 26 mm NaHCO_3_, 3 mm KCl, 2 mm CaCl_2_, 1.25 mm NaH_2_PO_4_, 1 mm MgSO_4_, 10 mm glucose, saturated with 95% O_2_–5% CO_2_ (pH 7.4) with an addition of 9 mm Mg^2+^. Horizontal brainstem slices containing the ventral surface of the medulla oblongata (thickness ∼300 μm; the pia mater was kept intact and undisturbed during slicing) or coronal brainstem and cortical slices (thickness 250 μm) were cut using a vibrating-blade microtome and then incubated at room temperature for 1 h in a standard aCSF saturated with 95% O_2_–5% CO_2_.

### Biosensor recordings

Lactate release was measured using amperometric lactate biosensors (Sarissa Biomedical, Coventry, UK) placed in direct contact with the surface of the slice. A lactate biosensor uses lactate oxidase that in the presence of oxygen converts lactate to pyruvate and H_2_O_2_ (which is detected electrochemically). A dual recording configuration of a null sensor (lacking lactate oxidase) placed in an equivalent position along the lactate biosensor was used (Fig.1*A*). The null sensor serves as a control to determine whether any non-specific electroactive interferents were released and confounded the measurements. Null sensor currents were subtracted from the lactate biosensor currents. Biosensors were calibrated with 100 μm lactate before and after the recordings (Fig.1*A*). To convert changes in sensor current to changes in lactate concentration, the means of the initial and final calibrations were used. Recordings were made from the slice placed on an elevated grid in a flow chamber at 35°C. Hypoxic conditions were induced for 2 min by replacement of oxygen in the medium with nitrogen (perfusion of the chamber with aCSF saturated with 95% N_2_ and 5% CO_2_). Since the lactate biosensor requires oxygen to operate, decreases in O_2_ concentration reduce biosensor currents (Fig.1*A*). Therefore, peak lactate release induced by hypoxia was determined upon re-oxygenation (Fig.1*A*).

**Figure 1:**
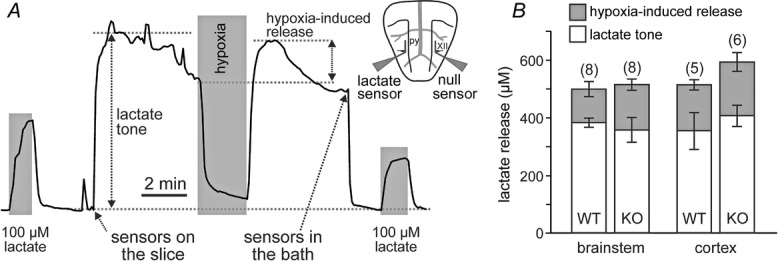
Tonic and hypoxia-induced release of lactate by the brainstem and cerebral cortex are not affected in conditions of MeCP2 deficiency *A*, representative example of changes in lactate biosensor current during calibration by application of l-lactate (100 μm), after biosensor placement in direct contact with the surface of the brainstem slice (revealing lactate tone), and in response to a hypoxic challenge (perfusion with aCSF saturated with 95% N_2_–5% CO_2_). Peak hypoxia-induced lactate release is measured upon re-oxygenation. Inset: schematic drawing of a horizontal brainstem slice illustrating dual recording configuration of lactate and null (control) biosensors placed on the ventral medullary surface. Difference in current between lactate and null biosensors was used to determine the amount of lactate release. py, pyramidal tract; XII hypoglossal rootlets; *B*, summary data showing tonic and hypoxia-induced release of lactate measured at the surface of the horizontal brainstem and coronal cortical slices of *MeCP2*-knockout (KO) and wild-type (WT) mice. Numbers in parentheses indicate sample sizes.

### Calcium imaging

Optical measurements of changes in [Ca^2+^]_i_ in brainstem astrocytes were performed using an upright Olympus FV1000 microscope (Olympus, Japan), equipped with 25× and 40× water immersion objectives. Regions for imaging were selected based on the distance from the pyramidal tracts, the boundary of the pons and the edge of the slice, targeting cells residing near the surface of the brainstem in locations corresponding to the chemosensitive retrotrapezoid nucleus of the rostroventrolateral medulla oblongata. [Ca^2+^]_i_ responses were visualised using a conventional Ca^2+^ indicator, Oregon Green-488 BAPTA-1, AM (OGB-1). Slices were incubated for 60 min at room temperature in 95 O_2_–5% CO_2_-saturated aCSF containing OGB-1 (10 μm) followed by 15 min incubation with sulforhodamine 101 (SR101, 30 μm) to aid identification of astroglial cells. Then, slices were washed for 15 min at room temperature in aCSF (saturated with 95% O_2_–5% CO_2_) for complete esterification of OGB-1. Imaging of Ca^2+^ responses was performed from the slice placed on an elevated grid in a flow chamber (volume 2 ml) at 35°C. The rate of perfusion was 4 ml min^−1^. The 488 nm argon laser line was used to excite OGB-1 fluorescence, which was measured using a bandpass filter of 505–550 nm. Illumination intensity was kept to a minimum (at 0.5–0.7% of laser output). SR101 fluorescence was excited at 514 nm, and emitted fluorescence at 641–716 nm was recorded. Hypercapnic conditions (respiratory acidosis) were induced by perfusion of the chamber with aCSF saturated with 90% O_2_–10% CO_2_. Fluorescence intensities in the regions of interest (ROIs) corresponding to astroglial somas were measured and background level was subtracted. On average responses of ∼10 labelled cells per slice were captured and analysed.

### Data analysis

Lactate biosensor measurements were processed using a 1401 interface and analysed using Spike2 software (Cambridge Electronic Design Ltd, Cambridge, UK). Built-in Olympus analysis software was used to analyse the results of the imaging experiments. Data are reported as means ± SEM. Datasets were compared using Wilcoxon’s signed rank test or Student’s *t* test for paired or unpaired data, as appropriate. Differences between groups with *P* values of less than 0.05 were considered to be significant.

## Results

Astrocytes are the only type of brain cell that store glycogen, and according to the evidence supporting the ‘lactate shuttle hypothesis’ produce and release lactate into the extracellular space to be taken up by neighbouring neurones (Pellerin & Magistretti, 1994). We first thought to determine whether the ‘metabolic’ function of astrocytes is compromised in conditions of MeCP2 deficiency by measuring the amount of lactate released by the brainstem slices of *MeCP2*-null and wild-type mice. For comparison, lactate release by cortical slices was also evaluated in order to determine whether MeCP2 deficiency is associated with global inability of astrocytes to release an appropriate amount of lactate. Tonic lactate release (lactate tone) was detected when lactate biosensors were placed in direct contact with the uncut ventral surface of the brainstem (Fig.1*A*) or coronal cortical slices of the *MeCP2*-null mice and their wild-type counterparts. No differences in the amount of lactate released by the brainstem slices were observed between preparations of the *MeCP2*-knockout (301 ± 27 μm, *n* = 19) and wild-type (310 ± 18 μm; *n* = 21) mice. Facilitated release of lactate in response to hypoxia was assessed in eight brainstem slice preparations of *MeCP2*-knockout and eight brainstem slice preparations of wild-type mice as well as in six cortical slice preparations of *MeCP2*-knockout and five cortical slice preparations of wild-type mice. No significant differences in the amount of lactate released in response to the hypoxic challenge were observed between brainstem and cortical slices of *MeCP2*-knockout and wild-type mice (Fig.1*B*).

Astrocytes which reside near the ventral surface of the medulla oblongata display [Ca^2+^]_i_ responses to chemosensory challenges and release gliotransmitters (predominantly ATP) which excite neurones of the brainstem respiratory network contributing to the adaptive increases in central respiratory drive (Gourine *et al*. 2010). We next determined whether CO_2_/pH sensitivity of medullary astrocytes is affected by MeCP2 deficiency.

Efficacy of SR101 labelling of brainstem astroglia was previously reported to be low (Schnell *et al*. 2012). Therefore, chemosensitive astrocytes of the ventral medulla oblongata were identified using several criteria: (i) labelling with SR101 (Fig.2*A*); (ii) Ca^2+^ response to hypercapnia (Fig.2*B*); (iii) lack of Ca^2+^ response to application of KCl (Fig.2*B*); and (iv) Ca^2+^ response to application of ATP (Fig.2*B*). In wild-type mice, respiratory acidosis (perfusion of the recording chamber with aCSF saturated with 90% O_2_–10% CO_2_) triggered robust elevations in [Ca^2+^]_i_ in astrocytes residing near the ventral surface of the medulla oblongata (Figs 3*A* and 4*A*). The magnitude of CO_2_-induced [Ca^2+^]_i_ responses in ventral medullary astrocytes was markedly reduced in conditions of MeCP2 deficiency (Figs 3*B* and 4*A*). CO_2_-induced [Ca^2+^]_i_ responses were equally suppressed in medullary astrocytes of both strains of *MeCP2*-null mice (*Bird* and *Coyle*, Fig.3*B*). Ca^2+^ responses triggered by application of exogenous ATP were not affected (Fig.3*B*, left panel).

**Figure 2:**
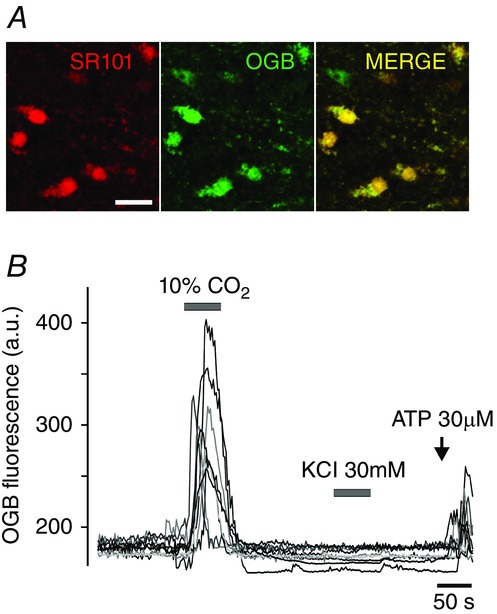
Chemosensitive astrocytes of the ventral medulla oblongata in mice *A*, putative astrocytes residing near the ventral surface of the medulla oblongata loaded with Ca^2+^ indicator Oregon Green-488 BAPTA-1, AM (OGB) and identified by labelling with sulforhodamine 101 (SR101). Scale bar, 25 μm; *B*, CO_2_/pH-sensitive astrocytes of the ventral medulla oblongata of a wild-type mouse identified by a [Ca^2+^]_i_ response to the respiratory acidosis (perfusion of the chamber with aCSF saturated with 90% O_2_–10% CO_2_), lack of Ca^2+^ response to application of KCl, and Ca^2+^ response to application of ATP.

**Figure 3:**
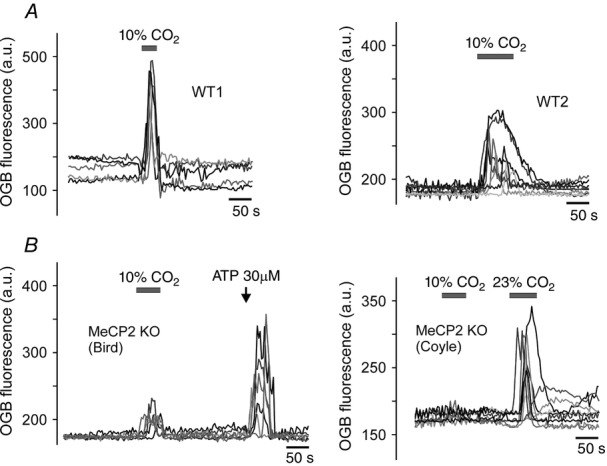
CO_2_-chemosensitivity of medullary astrocytes is compromised in conditions of MeCP2 deficiency *A*, representative examples of CO_2_-evoked [Ca^2+^]_i_ responses in astrocytes recorded in brainstem slice preparations of two individual wild-type (WT) mice; *B*, representative examples of CO_2_-evoked [Ca^2+^]_i_ responses in astrocytes recorded in brainstem slice preparations of *Bird* (left) and *Coyle* (right) strains of *MeCP2*-knockout (KO) mice.

**Figure 4:**
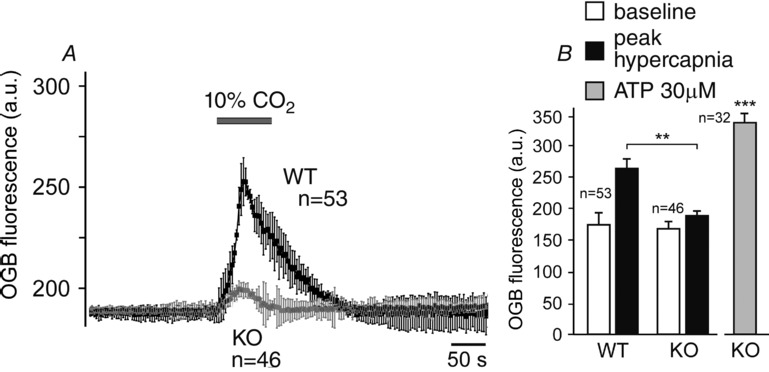
Summary data illustrating the effect of MeCP2 deficiency on CO_2_-induced Ca^2+^ responses of ventral medullary astrocytes *A*, time course of [Ca^2+^]_i_ changes induced by respiratory acidosis (perfusion of the chamber with aCSF saturated with 90% O_2_–10% CO_2_) in astrocytes recorded in brainstem slices of *MeCP2*-knockout (KO) mice and their wild-type (WT) counterparts; *B*, summary data of basal OGB fluorescence and peak CO_2_-induced increases in OGB fluorescence in astrocytes recorded in brainstem slices of *MeCP2*-knockout and wild-type mice. Values of *n* indicate the numbers of CO_2_-sensitive astrocytes recorded in 8 knockout and 8 wild-type brainstem slice preparations. **Significant difference in peak CO_2_-evoked response between wild-type and *MeCP2*-knockout astrocytes (*P* < 0.01). ***Significant difference in OGB fluorescence at the peak of the ATP-induced response compared to the baseline (*P* < 0.001).

In astrocytes recorded in slice preparations of *MeCP2*-knockout animals which failed to respond to a standard chemosensory challenge (10% CO_2_), [Ca^2+^]_i_ responses were triggered when severe hypercapnia/acidosis was applied (Fig.3*B*, right panel). These data demonstrate that MeCP2 deficiency reduces the ability of medullary astrocytes to sense changes in 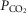/[H^+^]. Preserved Ca^2+^ responses to activation of ATP receptors suggests that the mechanisms underlying Ca^2+^ recruitment from the intracellular stores and/or Ca^2+^ entry are not affected by MeCP2 deficiency.

## Discussion

Rett syndrome – a prototypical neurological disorder caused by MeCP2 deficiency – is associated with a severely disordered breathing pattern (Southall *et al*. 1988; Julu *et al*. 2001; Weese-Mayer *et al*. 2006; De Felice *et al*. 2014) and reduced ventilatory CO_2_ sensitivity (Zhang *et al*. 2011; Bissonnette *et al*. 2014). In a mouse model of Rett syndrome (*MeCP2* gene knockout), re-introduction of the *MeCP2* gene selectively in astrocytes rescues normal respiratory phenotype (Lioy *et al*. 2011), suggesting that astroglial dysfunction underlies the development of the disease. Moreover, in mice selective and inducible deletion of *MeCP2* in astrocytes leads to a dramatically reduced ventilatory sensitivity to CO_2_ (Bissonnette *et al*. 2012), indicating that the respiratory neural circuits, including putative central chemosensitive neurones, are unable to generate appropriate CO_2_-sensitive respiratory drive when astrocytes are devoid of MeCP2. These data support the hypothesis of a key role played by astrocytes in the mechanisms of central CO_2_ chemosensitivity (Gourine *et al*. 2010). However, the mechanisms underlying reduced ventilatory CO_2_ sensitivity in conditions of MeCP2 deficiency are not known. Since astrocytes provide neural circuits with structural and metabolic support (metabolic function) and also have a signalling role, it remains unclear which of these two key astroglial functions is affected.

In the present study we found no differences in tonic and hypoxia-induced release of lactate measured from the ventral surface of the medulla oblongata or cerebral cortex in brain slices of *MeCP2*-knockout and wild-type mice. However, Ca^2+^ responses of ventral medullary astrocytes of *MeCP2*-knockout animals evoked by standard experimental chemosensory challenge (10% CO_2_) were dramatically reduced, while Ca^2+^ elevations induced by application of the exogenous ATP were preserved. In conditions of MeCP2 deficiency, robust Ca^2+^ responses in medullary astrocytes were only elicited when severe chemosensory challenge (>20% CO_2_) was applied.

Preserved lactate tone and unaltered release of lactate in low oxygen conditions in preparations of *MeCP2*-null mice suggest that metabolic function of astrocytes in providing neurones with the energy substrate in the form of lactate is not affected in the absence of MeCP2 function. This is somewhat surprising considering the variety of astroglial gene pathways controlled by this nuclear protein (Yasui *et al*. 2013; Forbes-Lorman *et al*. 2014). The ability of MeCP2 deficient brain tissue to release an appropriate amount of lactate might be an important factor in preventing or delaying widespread neuronal death and allowing recovery of synaptic function after acute tissue hypoxia (Schurr *et al*. 1997*a*,*c*,*b*), despite significant oxidative stress associated with recurrent episodes of intermittent hypoxia (De Felice *et al*. 2012, 2014). Indeed, it is not uncommon for Rett syndrome patients to experience episodes of severe oxygen desaturation (S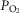 ≤ 80%) (Andaku *et al*. 2005; Hagebeuk *et al*. 2012; Carotenuto *et al*. 2013), yet post-mortem reports revealed no evidence of widespread neuronal degeneration (Cornford *et al*. 1994; Armstrong, 1995; Bauman *et al*. 1995). This is also supported by the evidence demonstrating that acute re-expression of *MeCP2* in adult knockout mice rescues the overt neurological phenotype, indicating that MeCP2 deficiency is associated with limited neurodegeneration (Guy *et al*. 2007). Interestingly, in patients with Rett syndrome, elevated cerebrospinal fluid (CSF) level of lactate positively correlates with respiratory dysfunction but not with other neurological features (Matsuishi *et al*. 1992, 1994; Lappalainen & Riikonen, 1994). The data reported here suggest that the high levels of lactate in the CSF of patients with Rett syndrome may reflect enhanced astroglial release in response to recurrent episodes of hypoxia that inevitably accompany the disordered breathing pattern associated with this condition (the significance of high CSF lactate in Rett patients in the context of the findings obtained in the present study is discussed below).

The results of the present study also show that MeCP2 deficiency dramatically reduced the ability of brainstem astrocytes to sense changes in 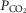/[H^+^]. Astrocytes are not electrically excitable and their signalling function is governed by changes in intracellular [Ca^2+^]. We previously demonstrated high sensitivity of medullary astrocytes to physiological changes in pH (Gourine *et al*. 2010). Astrocytes respond to hypercapnia/acidosis with robust elevations in [Ca^2+^]_i_, leading to the release of ATP, which propagates astrocytic activation and excites neurones of the brainstem respiratory network, contributing to the adaptive increases in central respiratory drive (Gourine *et al*. 2005, 2010; Kasymov *et al*. 2013). Astroglial CO_2_/pH sensitivity has been incorporated in the current models of brain respiratory chemosensitivity, centred on a group of highly pH sensitive neurones of the medullary retrotrapezoid nucleus (RTN), which are proposed to play the most significant role, with neighbouring astrocytes providing an additional 20–30% of the chemosensory drive to breathe (Guyenet, 2014).

This study contributes to the body of evidence suggesting that the role of astrocytes in the central nervous mechanisms linking changes in brain parenchymal 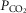/[H^+^] and central respiratory drive is greater than was previously appreciated. First, sensitivity of RTN neurones to changes in pH (as assessed in the organotypic brainstem slice preparation) is, to a large extent, mediated by prior release of gliotransmitter(s), primarily ATP (Gourine *et al*. 2010). Other experiments conducted in acute slices estimated that ATP actions contribute some 30% to the overall response of RTN neurones to respiratory acidosis (Wenker *et al*. 2012). Second, specific genetic targeting and silencing of RTN neurones reduces phrenic nerve response to systemic hypercapnia *in vivo* by ∼30% (Marina *et al*. 2010) – the effect similar in magnitude to that observed following blockade of ATP receptors within the region (Gourine *et al*. 2005; Wenker *et al*. 2012). Third, in mice astrocyte-specific inducible deletion of *MeCP2* results in a dramatically reduced ventilatory sensitivity to CO_2_ (Bissonnette *et al*. 2012), the effect being similar to that observed in animals with global MeCP2 deficiency (Zhang *et al*. 2011; Bissonnette *et al*. 2014). If astrocytes contribute only a small fraction of the overall respiratory response to systemic hypercapnia, then intact functional chemosensitive RTN neurones would be expected to mount an appropriate ventilatory response regardless of astroglial involvement (especially when we consider that the metabolic function of astrocytes does not appear to be affected by MeCP2 deficiency). Finally, the data reported here demonstrate that astroglial sensitivity to changes in 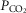/[H^+^] is dramatically reduced in the absence of MeCP2, the effect being consistent with associated respiratory phenotype and the hypothesis of a prominent role played by astroglia in the mechanisms of central respiratory CO_2_ chemosensitivity. It was possible to trigger [Ca^2+^]_i_ responses in MeCP2-deficient astrocytes by application of a severe hypercapnic stimulus (>20% CO_2_) suggesting a shift in sensitivity, although we did not investigate this systematically. Interestingly, shift in ventilatory CO_2_ sensitivity has also been demonstrated in *MeCP2*-null mice (Zhang *et al*. 2011; Toward *et al*. 2013; Bissonnette *et al*. 2014).

The mechanisms of intracellular Ca^2+^ recruitment evoked by activation of purinoceptors do not appear to be affected, as the responses to the exogenously applied ATP were preserved in astrocytes of *MeCP2*-null mice. These data suggest that reduced CO_2_ sensitivity in conditions of MeCP2 deficiency is upstream from astroglial release of ATP (which under normal conditions propagates Ca^2+^ excitation among ventral medullary astrocytes; Gourine *et al*. 2010). However, the mechanisms of CO_2_/pH sensitivity of astrocytes remain unknown. A number of putative mechanisms have been proposed including involvement of (i) certain ion channels (e.g. pH sensitive inward rectifier K^+^ channels – Kirs) (Mulkey & Wenker, 2011), (ii) a molecular CO_2_ sensor (e.g. connexin 26) (Huckstepp *et al*. 2010*a*,*b*), and (iii) an electrogenic Na^+^/HCO_3_^−^ cotransporter (Mulkey & Wenker, 2011).

Of these mechanisms MeCP2 deficiency has been shown to alter expression of the pH sensitive Kir4.1 channels in the locus coeruleus (Zhang *et al*. 2011) – one of the functional respiratory CO_2_ chemosensitive brainstem areas. It was suggested that overexpressed Kir4.1 subunit may favour formation of homomeric Kir4.1 channels, which are less sensitive to changes in pH than heteromeric Kir4.1–Kir5.1 (Zhang *et al*. 2011). Kir4.1–Kir5.1 heteromeric channels are expressed by astrocytes and have been proposed by some studies to underlie astrocytic CO_2_/pH sensitivity (Wenker *et al*. 2010; Mulkey & Wenker, 2011). Signalling molecules released by activated astrocytes (ATP and lactate) have a significant impact on the activity of locus coeruleus neurones (Harms *et al*. 1992; Tang *et al*. 2014), suggesting that reduced chemosensory responses of these neurones in conditions of MeCP2 deficiency (Zhang *et al*. 2011) may also be secondary to impaired CO_2_ sensitivity of local astrocytes. Indeed, in astrocytes Kir4.1 expression is regulated by DNA methylation throughout development, and therefore MeCP2 deficiency may lead to Kir4.1 overexpression (Nwaobi *et al*. 2014). However, more recent studies demonstrated that Kir4.1 expression is reduced in cortical astrocytes of heterozygous MeCP2 deficient female mice (Cuddapah *et al*. 2013) and the earlier evidence argues against any significant role of Kir4.1–Kir5.1 heteromeric channels in the mechanisms of central respiratory chemosensitivity (Trapp *et al*. 2011).

One potential mechanism which may underlie reduced CO_2_ sensitivity of MeCP2 deficient astrocytes may be related to the disruption of brain-derived neurotrophic factor (BDNF)-mediated signalling (Amaral *et al*. 2007). MeCP2 is an important regulator of BDNF transcription (Chen *et al*. 2003; Martinowich *et al*. 2003). BDNF protein levels are reduced in conditions of MeCP2 deficiency (Chang *et al*. 2006; Wang *et al*. 2006), while stimulation of BDNF receptors improves respiratory pattern in MePC2 null mice (Schmid *et al*. 2012; Kron *et al*. 2014). Part of the BDNF signalling pathway involves activation of certain transient receptor potential channels (TRPCs) (Amaral *et al*. 2007). Noteworthy is a recently described TRPA1 channel that plays an important role in generation of Ca^2+^ transients in astrocytes (Shigetomi *et al*. 2013). Together, these pieces of evidence suggest that disruption of BDNF- and TRPC-mediated Ca^2+^ homeostasis and signalling in astrocytes may underlie reduced Ca^2+^ responses to CO_2_ in medullary astrocytes of *MeCP2*-null mice.

The combination of preserved tonic and hypoxia-induced lactate release and markedly reduced CO_2_ sensitivity of medullary astrocytes could underlie severely disordered breathing observed in Rett syndrome. Cheyne–Stokes-like breathing pattern characterised by recurrent central apnoeas followed by periods of hyperventilation is frequent in Rett syndrome patients (Julu *et al*. 2001) and is also observed in mouse models (Bissonnette & Knopp, 2008; Abdala *et al*. 2010). Recurrent apnoeas followed by periods of hyperventilation are associated with chronic hypoxaemia and hypocapnia, as O_2_ saturation does not fully recover from apnoeas, and hyperventilation washes out CO_2_ (De Felice *et al*. 2014). Our data suggest that the acute bouts of severe hypoxaemia and hyperventilation would lead to the increased levels of brain lactate, as was demonstrated by measurements of high CSF lactate in Rett syndrome patients (Matsuishi *et al*. 1992, 1994; Lappalainen & Riikonen, 1994). This, in turn, would contribute to brain parenchymal acidification, which would facilitate hyperventilation, washing out more CO_2_. Increased CO_2_ apnoeic threshold and reduced CO_2_ sensitivity would favour prolonged apnoeas.

In summary, the data obtained in the present study suggest that MeCP2 deficiency is associated with a dramatically reduced sensitivity of medullary astrocytes to changes in 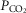/[H^+^]. Metabolic function of astrocytes in providing neurones with readily available metabolic substrate in the form of lactate does not appear to be affected. Taken together with the evidence of a severely blunted ventilatory response to CO_2_ in conditions of inducible deletion of *MeCP2* specifically in astrocytes (Bissonnette *et al*. 2012), these data support the hypothesis of an important role played by astroglia in the mechanisms of central respiratory CO_2_/pH chemosensitivity.

## Abbreviations

aCSF: artificial cerebrospinal fluid
BDNF: brain-derived neurotrophic factor
CSF: cerebrospinal fluid
KO: knockout
MeCP2: methyl-CpG-binding protein 2
OGB: Oregon Green-488 BAPTA-1 AM
SR101: sulforhodamine 101
TRPC: transient receptor potential channels
WT: wild-type
